# Distortion in time perception as a result of concern about appearing biased

**DOI:** 10.1371/journal.pone.0182241

**Published:** 2017-08-08

**Authors:** Gordon B. Moskowitz, Irmak Olcaysoy Okten, Cynthia M. Gooch

**Affiliations:** 1 Lehigh University, Psychology Department, Bethlehem, PA, United States of America; 2 Lehigh University, Psychology Department, Bethlehem, PA, United States of America; 3 Temple University, Program in Neuroscience, Philadelphia, PA, United States of America; Duke University, UNITED STATES

## Abstract

Two experiments illustrate that the perception of a given time duration *slows* when white participants observe faces of black men, but only if participants are concerned with appearing biased. In Experiment 1 the concern with the appearance of bias is measured as a chronic state using the external motivation to respond without prejudice scale (Plant & Devine, 1998). In Experiment 2 it is manipulated by varying the race of the experimenter (black versus white). Time perception is assessed via a temporal discrimination task commonly used in the literature. Models of time perception identify arousal as a factor that causes perceived time to slow, and we speculate that arousal arising in intergroup interactions can alter time perception.

## Introduction

Implicit bias is typically defined as stereotyping and prejudice that impacts people outside of awareness and without conscious intent [1]. It arises when a stereotype or an attitude (or both) is triggered outside of awareness by a cue associated with a social group. These “primed” stereotypes and affective responses manifest as bias toward that group by shaping how we categorize, where we allocate attention, the types of judgments and inferences we form, the expectations and standards we set, what we consider valid and veridical, how we feel, our approach and avoidance tendencies, and how we more generally act [2]. Does it manifest in something as basic, and low-level, in our psychological experience as how we perceive?

A relatively small body of empirical work examines bias in perception due to social stimuli [3–11]. These are largely focused on bias to visual perception (by race, but also by culture, expectations, norms, values and goals). For example, in the domain of race, Eberhardt et al. [6] had participants view a racially ambiguous face. For some participants the face was labeled “black,” for others it was labeled “white”. The task was to draw the face while looking at it. The facial features and skin tone of the drawings made of the face labeled “black” were more stereotypic (e.g., darker skin) than those labeled “white”. Such stereotypic drawings are evidence of the perceiver’s biased perception of the face that served as the model.

Just as what we see is distorted during the perceptual process by race, *time perception* may be distorted by race. Prior research outside the domain of race has shown that emotionally-laden stimuli in the form of angry facial expressions can distort time perception [12–14]. The nature of this distortion is an overestimation of time–time slows. To date, only one published set of experiments examines the impact of race as a biasing influence in time perception [15]. That research found that white participants perceived faces of black men to be of a longer duration than faces of white men, but only among participants who found race to be a threatening topic.

The current research examines the question of whether race impacts time perception at the level of milliseconds to seconds. Specifically, it examines the influence of race on the perception of the duration of face presentation. We argue that because arousal is known to slow the perception of time [16–18], individuals who associate arousal with race will have time perception distorted, slowed. In a first experiment we posit that white participants who have strong chronic concerns about *appearing* biased to African Americans will exhibit a time perception bias when perceiving faces of black men. This experiment is an exact replication of an earlier experiment [15], with an additional condition added to examine the impact of a heightened egalitarian goal as a mitigating influence on the bias. White individuals in the United States who associate arousal with race are identified in this experiment using the external motivation to control prejudice scale (EMCP) that had been used in the earlier experiment [15]. The nature of this scale, and why it is believed to assess a relationship between race and arousal [19], is described below.

In a second experiment we manipulated whether white participants were placed in an experiment with a black experimenter to test the hypothesis that a cross-race interaction will trigger arousal even in White participants who do not have strong chronic concerns about *appearing* biased to African Americans. We posit that when a white participant (who is not chronically high in EMCP) is placed in a racially defined situation, arousal will be triggered and this will slow time perception to faces of black men.

### Influences on time perception

**Fig 1:**
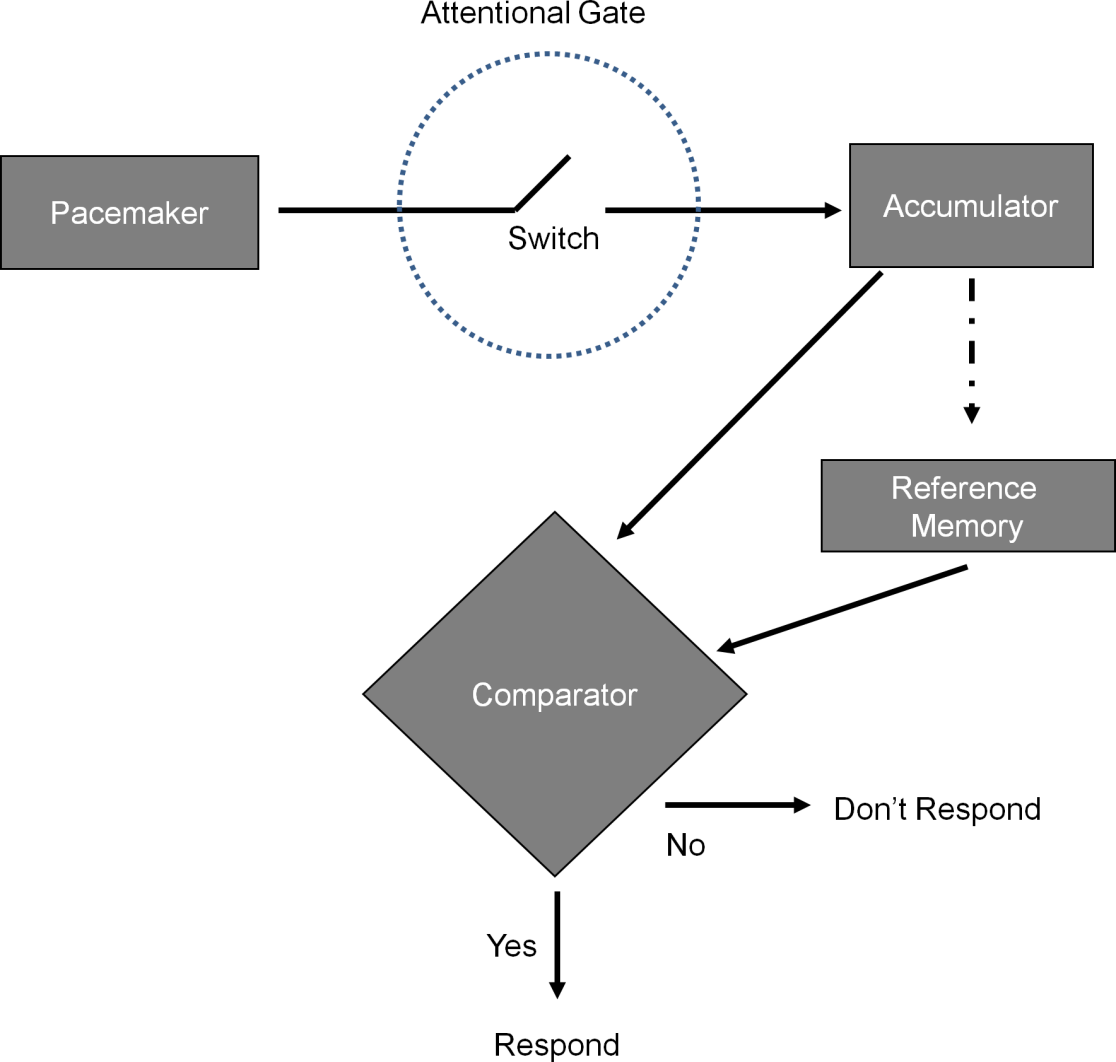
Scalar Expectancy Theory (SET) model of time perception. Adapted from Church, 1984. To summarize: a pacemaker releases isochronous signals, which are collected by an accumulator function when attention is directed to time passing. The accumulator values can be compared to a reference memory for accumulator values from a previous exposure to the time interval, to determine whether the currently timed interval has lasted a similar duration (comparator function). If the difference between the value from reference memory and the current accumulator value is below a given threshold, the decision is made that the time interval is equal to the memory for a standard. The model was originally developed to explain animal behavior in a fixed interval/peak interval paradigm, but has been applied to human timing as well.

A dominant model of time perception (Fig 1) argues that time perception occurs through several processes [16]. First, a *pacemaker* emits pulses at a constant rate. Second, when paying attention to the duration of a stimulus, an *attentional switch* closes, allowing the pulses to reach an *accumulator*. Third, the accumulator keeps count of the pulses emitted by the pacemaker. Fourth, current accumulator values are compared to a reference memory for the number of pulses that previously marked the learned duration (such as a half second). Time perception is based on a representation of the subjective count of the pulses that accrued in the accumulator as it compares to the reference memory for the number of pulses comprising that duration. (e.g., a representation of the pulses indicating a half second). Finally, a comparator engages in monitoring of the discrepancy between accumulated pulses and a threshold level of pulses defined by the stored representation. When the comparator reveals the difference between accumulator value and reference memory value drops below a threshold, one perceives that the duration being tracked has transpired.

Arousal, and attention to the passage of time, can distort the perception of time by impacting these processing stages. High levels of arousal (induced through noxious noise, drugs, physical exertion, facial expressions, etc.) speeds up the internal clock by causing more pulses in the same amount of time. Thus, the accumulator value that matches a reference memory arrives sooner. A duration of 500 ms might feel like it has arrived after only 250 ms, making the actual time of 500 ms feel longer. That is, *time slows*—the world outside seems to be moving slower [13–14][20]. A given time interval is perceived as longer.

Attention to time’s passage has similar consequences on perception of a stimulus’ duration. With focus on time (such as when watching the clock when bored), time slows. According to the model, the attentional switch closes and opens as vigilance waxes and wanes, and when focused on a stimulus’ duration, vigilance to time is heightened and the switch is closed more than it typically would be. With the switch closed, more pulses get to the accumulator during a given duration. Thus, the correct number of pulses to match the standard accrues more quickly, and the reference value is reached before the target duration has actually transpired. This causes the experience of the standard time as arriving sooner, making time seem to slow; the length of a given duration is overestimated [21].

### The motivation to avoid appearing biased

Cross-race interactions can be arousing. The more one is concerned with bias, the more taxing and arousing cross-race interactions become [22–25]. This manifests in malignant forms of cardiovascular reactivity [26], nonverbal communication problems such as avoiding eye contact, and setting greater interpersonal distance [27–28], showing preference to ingroups via greater cooperation, friendship seeking, and partner selection [29–31], self-regulation failures [32–34], and terminating interactions sooner [35–37].

Even striving to be egalitarian, and to not be biased, does not necessarily prevent one from the experience of such arousal. Plant and Devine [19] posited two distinct goals that can potentially be associated with egalitarianism. These two goals have disparate effects on the experience of arousal during cross race interactions. It is even the case that one manner of striving to be unbiased actually exacerbates arousal and can unintentionally heighten bias.

Individuals with goals to act fair/unbiased that emerge from *internal sources of motivation* (concern for equality, passion for social justice, desire for friends from diverse backgrounds and cultures, etc.) view members of stereotyped outgroups as opportunities that afford one the chance to pursue a cherished goal [38–40]. Such interactions are not anxiety provoking for people high in the internal motivation to control prejudice, but rather focus their attention on goal-relevant people and stimuli [41–42].

However, for individuals whose goals to act in an egalitarian way stem from external sources (concern with: others opinions, not doing the socially incorrect thing, not wanting to seem biased, etc.), cross-race interactions become an arousing chance for bias and social incompetency to be discovered [19] [24] [43]. It is precisely this type of externalized egalitarian goal, and its associated racial arousal, that is measured by the external motivation to control prejudice (EMCP) scale. Prior research has established that although EMCP is not associated with domain-general measures of social desirability, it does predict high arousal during intergroup responses [38] [43]. People who are high in EMCP have heightened arousal responses, heightened self-consciousness, and heightened focus on expressing bias when contemplating and engaged in cross-race interactions–even when white participants are simply presented with faces of black men [33] [38] [44]. Anything that triggers race for participants high in EMCP has been shown to also trigger arousal and bias associated with that arousal (such as directed attention to the arousing stimulus).

### Race and perceptual experience

Models of time perception dictate that when individuals are aroused, the perception of time (duration) for a target stimulus slows. Research on motivation to control prejudice illustrates that high EMCP is defined in white American participants by an arousal linked to the concern with the appearance of being biased toward African Americans. Thus, our hypothesis is that time perception is impacted by race, but only among people who are concerned with the appearance of bias. This should occur for people whose basis for egalitarian action is externally motivated (high EMCP). Time perception being impacted by race will be manifested in estimates of stimulus duration–the duration of black faces will feel longer than the duration of white faces that are presented for the same length of time. The perceptual distortion, therefore, should not emerge when people do not experience arousal, such as when they are not chronically oriented to have external concerns with appearing biased.

However, it might be possible that the arousal that people high in EMCP experience can be ameliorated by temporary conditions that create egalitarian goals for which the striving to be non-prejudiced is internally located. That is, if we create a conflict between their chronic EMCP goals and their temporary internal egalitarian goals, the temporary and internal goals might drive responding. To explore this possibility we manipulated EMCP, but also independently introduced a goal priming procedure [45] that temporarily manipulated the strength of one’s internal egalitarian goals.

## Experiment 1

### Materials and methods

#### Procedure

Levels of EMCP [19] were recorded during an online testing session as part of registration for a voluntary participant pool. Between 2 to 8 weeks after the registration period participants were brought to the lab one at a time and asked to perform a temporal discrimination task on the computer. After providing signed consent they were brought to a small room with a single computer for the task. Task instructions were displayed on screen; the experimenter stayed in the room until participants indicated they understood the procedure. A procedure was used in which a stimulus was presented for a standard time, and participants were asked to judge whether a comparison stimulus (e.g., a face) appeared for a longer or shorter time than the standard stimulus. This response choice repeated across more than 200 stimulus displays (trials). Race of faces and the duration for which they were presented was manipulated as a within-participants variable across these many trials. Unbeknownst to participants goal primes were subliminally flashed prior to each trial of the discrimination task, thus manipulating goals between participants (egalitarian goal versus control condition). When the task finished, participants were informed by on-screen instructions to exit and meet the experimenter in an adjacent room (where they had signed their consent). They were then debriefed and thanked for their participation. This research involved human subjects and the protocol was approved by Lehigh University’s institutional review board. The work was carried out in accordance with the provisions of the World Medical Association Declaration of Helsinki.

#### Participants

Participants were 63 white students (37 women and 26 men) enrolled in the introduction to psychology course at Lehigh University, ranging in age from 18–22. Participation was voluntary and compensated with a credit toward a course requirement. Two participants’ responses could not be fit to the curve that was used to calculate the point of subjective equality that is explained below (as they gave the same response, e.g., “longer”, on most trials of the time perception task despite the varying durations of the stimuli they were meant to be judging). Because the unit of analysis that summarized their responses (the PSE) could not be calculated by the MATLAB formula we employed (see below), those two participants’ had no data to enter into our analyses, and hence their data were dropped in our analyses. The high EMCP group was 64% women, compared to the group of people not high in EMCP which was 56% women.

The sample size was determined by a power analysis using the software program “MorePower,” Version 6.0.1 [46]. The power analysis suggested that for an effect size between small to medium [15] (*η*_*p*_ = .1 and *η*_*p*_ = .2, α = .05 and power = .9, repeated measures: 1 (3-level), independent measures: 2 x 2) we would need between 32 to 64 participants. Thus, we decided to request 64 white participants from the participant pool and included as many of them in the experiment as we could recruit. Our stopping rule was to end data collection when we recruited 64 participants or ran out of participants in the pool eligible for the experiment (whichever came first).

#### Assessment of motivation to respond without prejudice

External motivation to respond without prejudice (EMCP) was assessed with a previously published and validated scale [19]. The scale consists of five questions assessed on a 9-point scale: Because of today’s PC (politically correct) standards I try to appear nonprejudiced toward Black people, I try to hide any negative thoughts about Black people in order to avoid negative reactions from others, If I acted prejudiced toward Black people I would be concerned that others would be angry with me, I attempt to appear nonprejudiced toward Black people in order to avoid disapproval from others, and I try to act nonprejudiced toward Black people because of pressure from others. The administration of the measure occurred several weeks prior to the experiment during registration for a voluntary participant pool maintained by the psychology department at Lehigh University. The registration included a battery of measures used among multiple experiments in the department. Participants with EMCP scores more than one standard deviation above the mean were labeled as high in EMCP, those remaining were labeled as not high in EMCP as in past research [15].

#### Time perception task

Time perception was assessed by a temporal-discrimination task in which participants responded to objects (i.e., geometric shapes) and faces. The method of constant stimuli was used; that is, on each trial, participants saw an image of a geometric shape in dark grey for a standard length of time (the standard duration) and then one image taken from a series of possible comparison stimuli; the second image, or comparison stimulus, appeared for different lengths of time. On some trials, the comparison stimuli were again geometric shapes (dark grey in color). On other trials the comparison stimuli were photographs of male faces selected from a widely-used database of faces that has been normed and pretested for perceived age and perceived emotional expression [47]. Half the faces were white men and half were black men, all photographed in black and white, and all were designated in prior research as having neutral emotional expressions.

On a given trial in this task, two images were presented sequentially on the computer monitor, first the standard stimulus and then the comparison stimulus. The participant was to indicate (by pressing buttons marked “shorter” and “longer” on the keyboard) whether the comparison stimulus had been presented longer than the standard stimulus had been presented. On every trial, the standard stimulus was one of several possible geometric shapes presented for 600 ms. Comparison stimuli were manipulated across trials to be either shapes or faces. The race of the faces was manipulated to be either black or white. The experiment comprised four blocks of trials; each block contained 21 trials with shapes as comparison stimuli, 21 trials with black male faces as comparison stimuli, and 21 trials with white male faces as comparison stimuli.

The duration of the comparison stimulus was manipulated across trials; comparison stimuli appeared for either 300, 380, 480, 600, 760, 960, or 1,200 milliseconds (ms). These durations were spaced logarithmically around the standard interval (i.e., 600 ms). Given the scalar property of time perception, such that discrimination is finer between two shorter intervals than between two longer intervals, this spacing promoted equal difficulty in discriminating shorter and longer intervals. Additionally, when plotting duration on the X axis and percentage of trials in which one responded “longer” on the Y axis, such spacing creates a more proportional sigmoid curve than would emerge using a linearly spaced set of durations. Paired with each duration in each block there were three shapes as comparison stimuli, three black male faces as comparison stimuli, and three white male faces as comparison stimuli. This created twelve pairings of each type of comparison stimulus type and each duration across the experiment.

This task typically produces high accuracy at the extreme long and short durations (300 and 1,200 ms); among the remaining durations, for which the correct answer is more ambiguous, accuracy is variable. When duration is plotted on the *x*-axis and the percentage of “longer” responses is plotted on the *y*-axis, the result is an S-shaped curve. When time slows, a comparison stimulus that appears for less than 600 ms is more likely to be inaccurately labeled as being “longer” than the standard time of 600 ms and a comparison stimulus that appears for longer than 600 ms is more likely to be accurately labeled as “longer”. This results in the sigmoid curve shifting left as a result of slowed time perception.

#### Goal manipulation

The temporary triggering of egalitarian goals was accomplished through a “goal priming manipulation” commonly used in the literature [45]. This task exposed participants either to words relating to egalitarian goals or to control words. Before each trial of the discrimination task a word was subliminally presented (shown for 15 ms). These two sets of words were drawn from prior research [41]. Half the participants had words related to egalitarianism subliminally flashed (equity, tolerance, fair, egalitarian, broadminded, justice), half had control words flashed that were matched for valence, length and frequency in the English language (warmhearted, responsible, courteous, humility, integrity, kindhearted).

### Results

**Fig 2:**
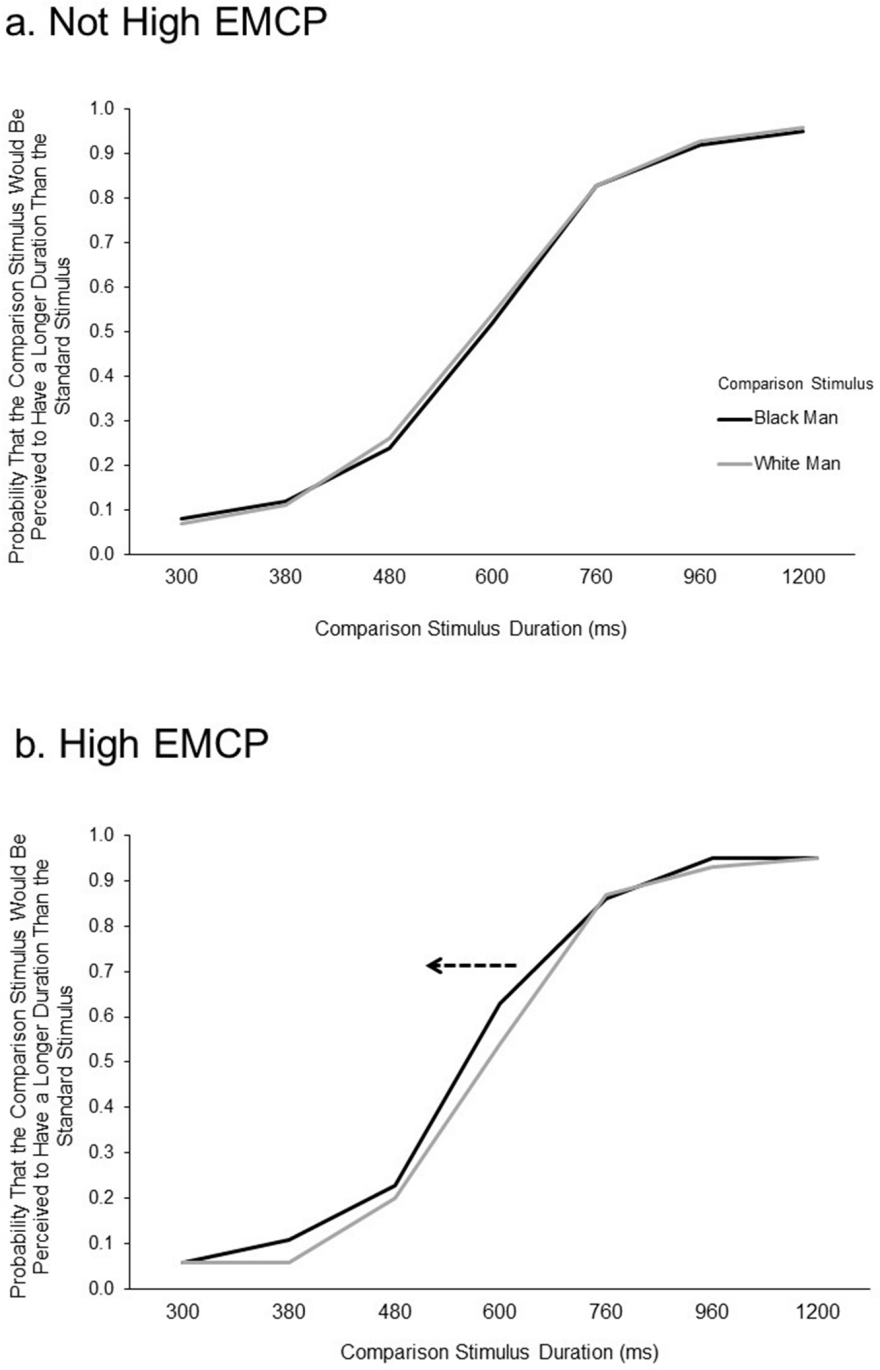
Experiment 1: Percent of “longer” responses at each stimulus duration for each stimulus type. Participants were divided on the basis of EMCP scores (see Methods). (a) Responses to pictures of Black Men and White Men in participants that did not score high on this measure. (b) Responses in participants that did score high on this measure. The dotted arrow in (b) signifies a shift of responses to Black Men in the High EMCP participants.

The hypothesis was that levels of external motivation to respond without prejudice would predict time distortion. The first evidence of time slowing when participants perceived faces of Black men was provided by the leftward shift of the S-shaped curve that resulted when duration was plotted on the *x*-axis and the percentage of “longer” responses was plotted on the *y*-axis. As illustrated in Fig 2A and 2B, the curve representing high-EMCP participants’ perception of duration of presentation of black male faces was shifted leftward compared with their curves for white male faces. However, participants who were not high in EMCP responded to the two types of faces in an almost identical fashion.

The primary measure of the slowing of time was the point of subjective equality (PSE). The PSE is a single period of time that represented the subjective perception of the comparison-stimulus duration being equal to the standard time of 600 ms. If time perception is slowing, the PSE will be lower (a shorter amount of time is mistakenly perceived as equivalent to the standard duration). Participants high in EMCP should perceive the PSE to be lower to faces of black men than to faces of white men. No such difference should emerge in participants not high in EMCP.

The PSE for each type of comparison stimulus is calculated by plotting each person’s duration judgments (longer/shorter) across the twelve trials of each type of comparison stimulus at each duration. The duration of stimulus presentation is plotted on the *x*-axis, and the proportion of trials in which a comparison stimulus was judged to be longer than the standard is plotted on the *y*-axis. The data points approximate an S-shaped curve and are fitted to the logistic function 1/10^−[*a* × (*x* − *b*)]^, in which *a* is an index of the slope, *b* represents the PSE, and *x* is the duration. Fits were made using the function lsqcurvefit in MATLAB (The MathWorks, Natick, MA). This function optimizes parameters of an equation (in this case, *a* and *b*) by minimizing the squared error between the data points and the curve produced by the equation. It uses the trust-region-reflective algorithm to make adjustments to the parameters to minimize sum of squares. This produces the PSE as a time interval signified by a point on the curve at which a participant is 50% likely to judge the comparison duration to be “longer” and “shorter” than the standard duration. That is, it is their perception of the standard time of 600 ms.

PSEs for each stimulus type (Black men, White men and Shapes) were calculated for each participant. This calculation provided three continuous PSE variables (one for each stimulus type). In addition, difference scores between (a) the PSE for faces of Black men and the PSE for shapes and (b) the PSE for faces of White men and the PSE for shapes were calculated for each participant. The difference score provided an index of the probability of misidentifying a time as longer than 600 ms for faces compared with shapes. That is, responses to shapes served as a baseline measure for the task against which responses to two different types of faces were evaluated. A PSE for a class of stimuli (such as faces of black men) lower than that found for control stimuli (geometric shapes) would indicate that time had slowed for perceiving that class of stimuli. Thus, the slowing or accelerating of time was indicated by PSE differences between stimuli of interest (white and black male faces) and control stimuli.

**Fig 3:**
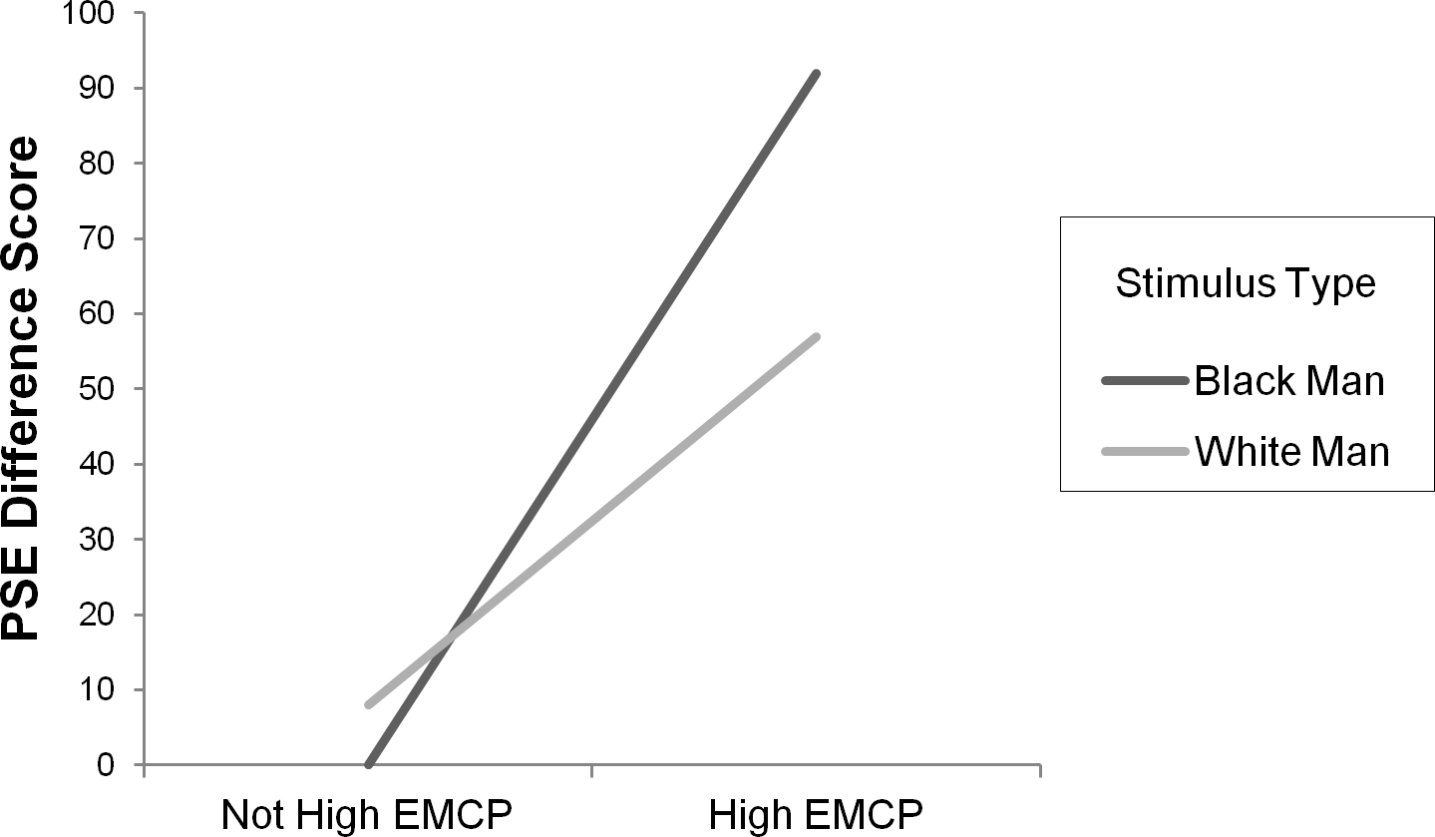
Experiment 1: PSE difference scores for each stimulus type. Data for each participant, for each stimulus type, were fit with a sigmoid curve and a resulting point of subjective equality (PSE) was calculated (see Methods). PSE Difference Score = (Object PSE–White Man PSE) OR (Object PSE—Black Man PSE). X axis represents different subject groups (EMCP score) and Y axis represents PSE difference score.

We conducted a Stimulus Type X EMCP X Goal Prime ANOVA with PSE difference score as the dependent variable and stimulus type, EMCP group and goal prime as the independent variables. There was no effect of the goal priming–no interactions or main effects with the subliminal goal prime emerged. Therefore, the effect of goal prime will no longer be discussed. However, the predicted interaction between EMCP and stimulus type emerged in this three-way ANOVA, *F*(1, 57) = 4.77, *p* < .04, *η*_*p*_ = .08 (and similarly when a Stimulus Type X EMCP ANOVA was conducted, collapsing across goal prime condition; *F*(1, 59) = 5.17, *p* < .03, *η*_*p*_ = .08). This is illustrated in Fig 3, which shows a lowering of the PSE for responses to black male faces relative to white male faces, but only for the high-EMCP group. When we performed the Stimulus Type X EMCP ANOVA using raw PSE scores (not difference scores) and controlled for shape PSE by entering it as a covariate, we found the same pattern, *F*(1, 58) = 4.42, *p* < .04, *η*_*p*_ = .07. The PSE was lower, as predicted, when the comparison stimulus was a black man’s face than when it was a white man’s face but this was only true for the high-EMCP group.

To further examine this pattern of means, a series of *t*-tests were conducted. As predicted, participants high in EMCP were found to have a lower PSE when responding to faces of black men (*M* = 568 ms) than when responding to faces of white men (*M* = 603 ms), *t*(21) = 2.14, *p* < .05, 95% CI [-68.77, -.98], Cohen's *d* = .461, as well as when responding to shapes (*M* = 660 ms), *t*(21) = -4.09, *p* < .01, 95% CI [-138.61, -.45.18], Cohen's *d* = -0.916. PSEs for faces of white men were also lower than shapes, *t*(21) = 3.40, *p* < .01, 95% CI [22.18, 91.86], Cohen's *d* = 0.767, yet not as much as faces of black men. For participants not high in EMCP, no difference was found in PSE when responding to black versus white faces (*M* = 599 ms and 591 ms respectively), black faces versus shapes (*M* = 599 ms and 614 ms respectively), or white faces versus shapes (*M* = 591 ms and 614 ms respectively), *p*’s > .45. Perhaps the most intuitive way to ask the question of time slowing is to examine if there is a PSE decrease when examining responses to black faces versus white faces. For participants high in EMCP there is a reliably greater drop in PSE when responding to black faces versus white faces (*M* = -35 ms) than for participants not high in EMCP (*M* = 8 ms), *t*(59) = 2.27, *p* < .03, 95% CI [5.10, 79.87], Cohen's *d* = 0.595 (with participants not high in EMCP experiencing no drop in PSE for black vs. white faces, *t*(38) = .717, *p* = .48, 95% CI [-13.88, 29.09], Cohen's *d* = 0.115.

Finally, we performed a regression analysis on PSE scores to further examine race-based distortion of time perception as a result of EMCP. PSE scores to shapes were entered first (since we are treating these as a form of baseline responding) with PSE scores to Black faces and White faces entered into the regression next at the same step. EMCP scores were expressed as a continuous variable. PSE for shapes did not predict EMCP significantly at the first step, *t*(59) < 1. However, adding PSEs for faces at the second step brought about a significant change in *R*^*2*^ by explaining an additional 10% of the variation in EMCP, *F*(2, 57) = 3.29, *p* <. 05. At this second step, a significant relationship emerged between EMCP and PSE for faces of black men, *β* = -.36, *t*(59) = -2.49, *p* < .02, 95% CI [-.087, -.009]. No relationship was found between EMCP and PSE for faces of white men or for shapes, *p’s* > .1 As hypothesized, we found that increases in EMCP reliably predicted a smaller PSE but only when participants were observing faces of black men. The greater a participant’s EMCP, the more he or she perceived time to slow when observing faces of black men.

In summary, the results show that for responses to faces of black men, relative to white men and shapes, short amounts of time are mistaken for longer ones. A black face is experienced as lingering longer. We refer to this phenomenon as time perception bias (TPB). But this is contingent on motivation. Participants perceive the time a face is on the screen differently for black versus white faces as a function of their level of external motivations relating to appearing prejudiced. The higher one’s external motivation to respond without prejudice, the shorter the duration an image of a black man needs to be on the screen for one to say it is 600 ms. The more concerned one is with controlling the bias, the more likely this bias in time perception is to emerge.

## Experiment 2

Are only specific types of individuals susceptible to this bias? In the first experiment the focus was on a set of people known from prior research to have arousal relating to appearing prejudiced. We hypothesized, and found, that for such individuals a bias to time perception could be triggered merely by seeing a face of an outgroup member (black men for white participants; and this was hypothesized as being due to the arousal such individuals are known to have chronically linked to race). But we further hypothesize that it is not only these types of individuals who would show TPB. Even an individual who was not possessing high level of external motivation to control prejudice can still experience arousal as it relates to race. For these individuals (the majority of people, white participants who are not one standard deviation above the mean in their EMCP score) such arousal would not be triggered merely by seeing a face. But being placed in a situation that was more fraught with racial anxiety could potentially create arousal in such white Americans, and thus bias to time perception would appear for any white person in such a situation. For this reason we chose to focus the second experiment exclusively on people who are not high in external motivation to control prejudice. Our goal was to see if the same effect could be induced in people without chronic levels of anxiety as it relates to race, and instead have the effect induced by the current situation. Of course, people high in EMCP, already showing distortion to time perception without the need of a situational inducement, would likely continue to show the bias (perhaps more strongly) under such conditions. But our concern in this experiment is not with such individual differences, but in the power of the situation to create time distortion. The situation we selected that we presumed would introduce arousal was an interaction.

Cross race interactions are stressful even among low-prejudiced people [22–26]. Such interactions provide an opportunity for one to appear prejudiced to other people [19][43] and to affirm personal concerns about whether one is truly egalitarian [32][48]. While people with high levels of EMCP have been shown to have arousal reactions merely at the thought of race, by the mere presentation of images of outgroup members [15][33][38][44], even people without such chronic tendencies can become worried about bias when placed in interactions with members of an outgroup. When contextual conditions create a heightened concern with appearing biased, and these conditions introduce race-based arousal, then any individual in that context would be expected to illustrate the same distortion to time perception. Interactions among two white people are less likely to trigger such arousal than an interaction among a white person and a black person, which for many white Americans introduces concerns about being perceived by others as prejudiced or saying something inappropriate [32].

It is hypothesized that the source of the perceptual bias is the experience of arousal, and in Experiment two this can arise from being placed in an interaction that is fraught with such concerns (turning away from the chronic concerns of focus in Experiment one). Therefore, in Experiment two, we manipulated whether the participants interacted with a black man or not. When the interaction is across race we expected that the arousal arising in such interactions (linked to the stereotype in the United States of black men as threatening) would be triggered [32]. This should impact how people react to faces of black men on the time perception task in a manner equivalent to the bias seen in Experiment one. In the control condition participants do not interact with a black person, but instead interact with a white woman. In the United States women are stereotyped as passive [49], and hence this interaction should not be arousing. Such a manipulation introduces the possibility that any subsequent effects are caused by the gender rather than race of the experimenter, but this possibility is easy to rule out with our data. If the effect is caused by gender, participants would respond equally with a time perception distortion to faces of men, both black and white. If caused by race the distortion would only be seen to faces of black men.

In Experiment 2 we sought to replicate the effect of time slowing illustrated in Experiment 1 by using an identical temporal discrimination procedure, but manipulated the threat/arousal potential of the experimenter. Following the social stereotypes, we had half of the participants interact with a black man during the experiment and half interacted with a white woman. Does a black experimenter cause arousal in white participants that will slow their perception of time to faces of black men even if the white participants are not high in EMCP? Using a temporal discrimination task, we examine if a cross-race interaction leads people who are not high in EMCP to perceive time to faces of black men in a manner similar to people who possess high levels of EMCP.

### Material and methods

#### Procedure

Several weeks before the experiment, as part of registration for a voluntary participant pool, ongoing levels of EMCP [19] were recorded during an online testing session. Between 2 to 8 weeks after the registration period participants who were less than one standard deviation above the mean in EMCP were brought to the lab one at a time and asked to perform a temporal discrimination task on the computer. The procedure and task was identical to Experiment 1 with a single exception. Rather than a single, white, female experimenter, participants were randomly assigned to one of two possible experimenters–a black man or a white woman. This research involved human subjects and the protocol was approved by Lehigh University’s institutional review board. The work was carried out in accordance with the provisions of the World Medical Association Declaration of Helsinki.

#### Participants

Participants were 77 white students enrolled in the introduction to psychology course at Lehigh University, ranging in age from 18–48. Participation was voluntary and compensated with a credit toward a course requirement. Three of those participants failed to perform the task appropriately (gave the same response on most of the trials) so their responses did not fit to the sigmoid curve and their PSE scores could not be calculated. Analyses were conducted with 74 participants.

The sample size was determined by a power analysis using the software program “MorePower” (Version 6.0.1) [46]. The power analysis suggested that for an effect size between small to medium (see Experiment 1 for the parameters; repeated measures: 1 (3 level), independent measures: 1 (2 level) we would need between 28 and 60 participants. We decided to request 80 white participants from the participant pool and included as many of them in the experiment as we could recruit, Our stopping rule was to end data collection when we recruited the 80 participants assigned to our experiment, or ran out of participants in the pool eligible for the experiment (whichever came first).

#### Assessment of motivation to respond without prejudice

External motivation to respond without prejudice (EMCP) was assessed as in Experiment 1. Participants with EMCP scores more than one standard deviation above the mean were not recruited for participation.

#### Time perception task

Time perception was assessed by a temporal-discrimination task identical to that used in Experiment 1.

#### Experimenter manipulation

The attempt to manipulate the temporary triggering of intergroup arousal was accomplished through the use of two different experimenters whose group memberships were associated with cultural stereotypes of threat versus passivity. Participants were greeted and run through the experiment either by a black man or a white woman.

### Results

**Fig 4:**
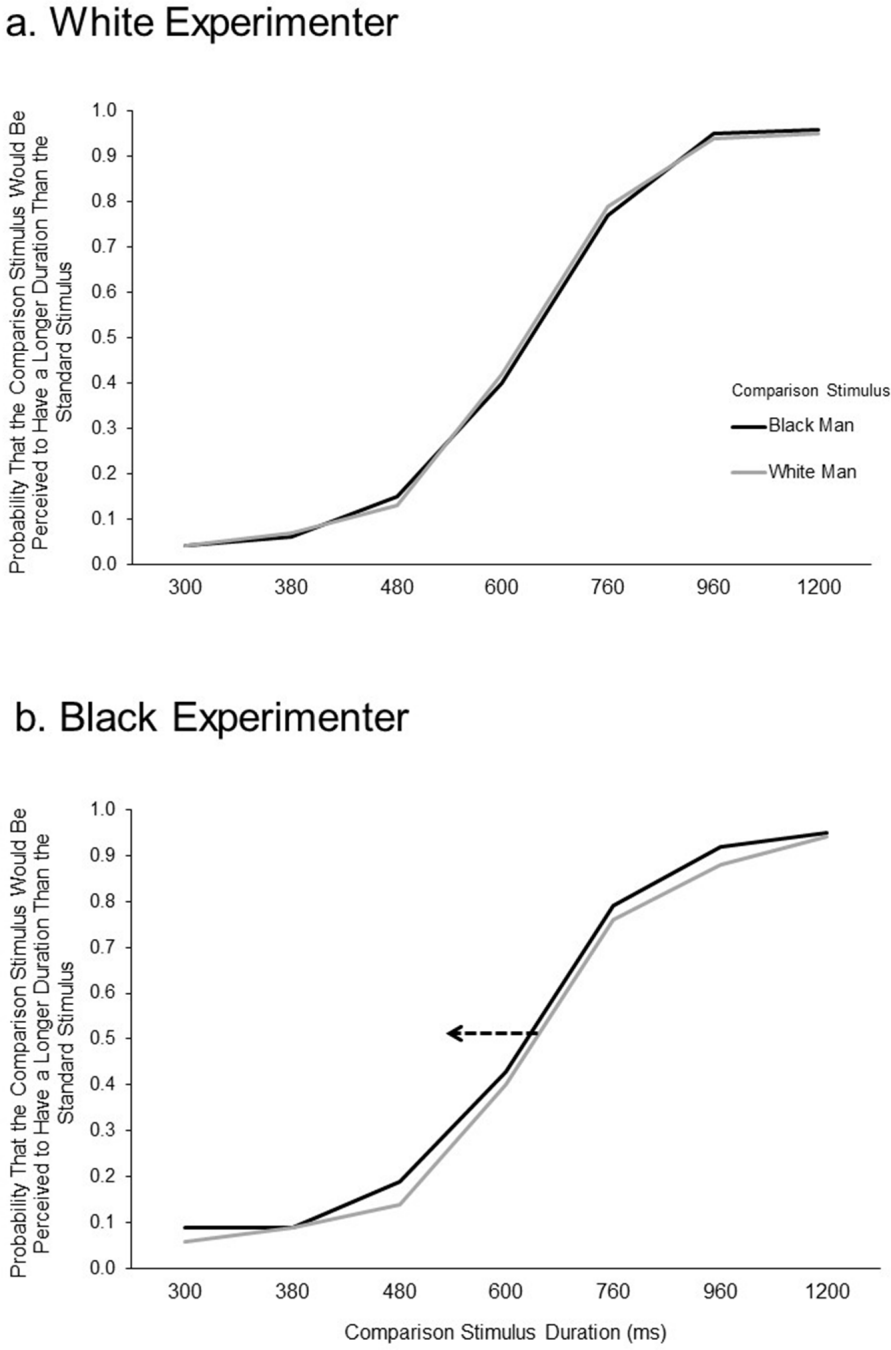
Experiment 2: Percent of “longer” responses at each stimulus duration for each stimulus type. Participants experienced either a White experimenter or a Black experimenter (see Methods). (a) Responses to pictures of Black Men and White Men in participants that participated with a White experimenter. (b) shows responses in participants that participated with a Black experimenter. The dotted arrow in (b) signifies a shift of responses to Black Men in the participants that participated with a Black experimenter.

The first evidence of time slowing when participants perceived faces of Black men was provided by the leftward shift of the S-shaped curve that resulted when duration was plotted on the *x*-axis and the percentage of “longer” responses was plotted on the *y*-axis. As illustrated in Fig 4A and 4B, the curve representing participants who interacted with a black experimenter was shifted leftward when comparing their perception of black male faces to their curves for white male faces. However, participants who interacted with a white woman as the experimenter responded to the two types of faces in an almost identical fashion.

The primary measure of the slowing of time was the PSE. Participants interacting with a black experimenter should perceive the PSE to be earlier to faces of black men than to faces of white men. No such difference should emerge in participants interacting with a white experimenter. The PSE was calculated for each participant in a fashion identical to Experiment 1. A PSE difference score between (a) the PSE for faces of Black men and the PSE for shapes and (b) the PSE for faces of White men and the PSE for shapes was also calculated as in Experiment 1.

**Fig 5:**
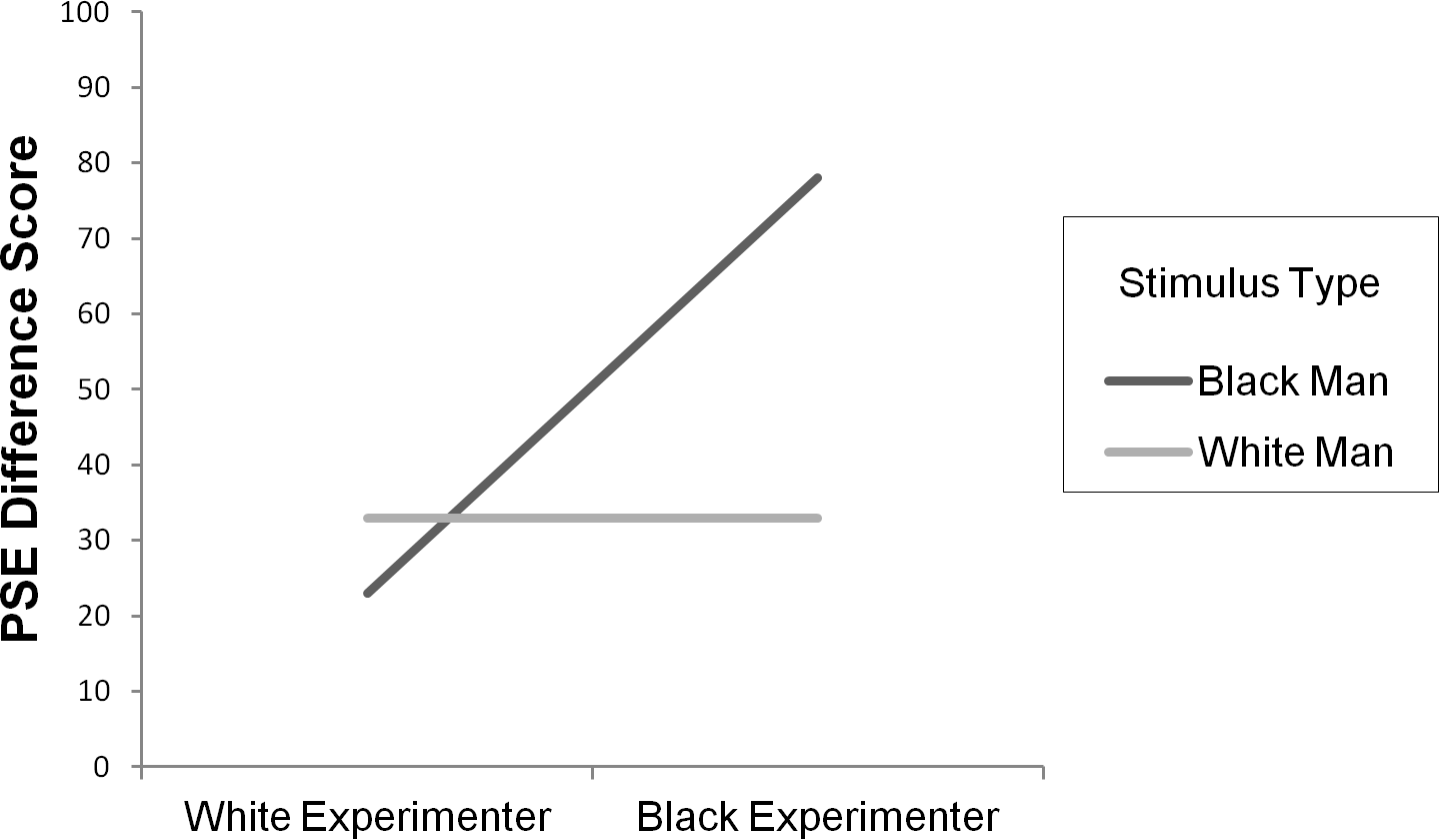
Experiment 2: PSE difference scores for each stimulus type. Data for each participant, for each stimulus type, were fit with a sigmoid curve and a resulting point of subjective equality (PSE) was calculated (see Methods). PSE Difference Score = (Object PSE–White Man PSE) OR (Object PSE—Black Man PSE). X axis represents different subject groups (experimenter group) and Y axis represents PSE difference score.

We conducted a Stimulus Type X Experimenter Type ANOVA with PSE difference score as the dependent variable and Stimulus Type and Experimenter Type as the independent variables. The predicted interaction between Experimenter Type and Stimulus Type emerged, *F*(1, 72) = 12.3, *p* < .01, *η*_*p*_ = .146. When we performed the analyses using raw PSE scores (not difference scores) and controlled for shape PSE by entering it as a covariate, we found the same pattern, *F*(1, 71) = 11.91, *p* < .01, *η*_*p*_ = .144. The PSE was lower, as predicted, when the comparison stimulus was a black man’s face than when it was a white man’s face, but only for participants who had been placed in a context where they interacted with a black experimenter. This is illustrated in Fig 5, which shows a lowering of the PSE for responses to black male faces relative to white male faces, but only for the participants who interacted with the black experimenter.

To further examine this pattern of means, a series of *t*-tests were conducted. As predicted, participants who interacted with the black experimenter were found to have a lower PSE when responding to faces of black men (*M* = 607 ms) than when responding to faces of white men (*M* = 652 ms), *t*(36) = -3.46, *p* < .01, 95% CI [-70.76, -18.41], Cohen's *d* = -0.568, as well as when responding to shapes (*M* = 685 ms), *t*(36) = -3.68, *p* < .01, 95% CI [-120.10, -34.68], Cohen's *d* = -0.611. Their PSEs for white men and shapes did not differ significantly, *t*(36) = -1.76, *p* = .09, CI [-70.64, 5.02], Cohen's *d* = -0.291. For participants who interacted with the white experimenter, no difference was found in PSE when responding to black versus white faces (*M* = 651 ms and 641 ms respectively, *t*(36) = 1.16, *p* = .25, CI [-7.71, 28.51], Cohen's *d* = 0.193) or black faces versus shapes (*M* = 651 ms and 674 ms respectively, *t*(36) = -1.81, *p* = .08, CI [-49.28, 2.81], Cohen's *d* = -0.299). Those participants’ PSEs for white faces were lower than shapes, *t*(36) = -2.31, *p* = .03, CI [-63.13, -4.14], Cohen's *d* = -0.380. Perhaps the most intuitive way to ask the question of time slowing is to examine if there is a PSE decrease when examining responses to black faces versus white faces. For participants who interacted with the black experimenter there is a reliably greater drop in PSE when responding to black faces versus white faces (*M* = -45 ms) than for participants who interacted with the white experimenter (*M* = 10 ms), *t*(72) = 3.50, *p* < .01, CI [23.70, 86.27], Cohen's *d* = 0.828.

## Conclusions

Research in social cognition provides important illustrations of implicit bias and the mechanisms through which such bias is manifested. But it is rarely concerned with bias to perceptual experience. How should race impact time perception? Like seeing and hearing, perceiving time is experienced by the individual as an objective reality since it is given no conscious attention–it proceeds outside awareness and governs our movement and decisions seamlessly. Yet, like other perceptual activity [3–11], time perception is open to influence.

We predicted that to white people in the U.S. time would slow when perceiving black men if they had arousal associated with black men. The experiments revealed this distortion in the perception of time for our white participants when perceiving images of black men when manipulating arousal in two distinct ways. In experiment one, the time perception bias (TPB) emerged among participants with strong external motivations relating to controlling prejudice and its expression. In experiment 2 the bias emerged when white participants were performing the time perception task under the supervision of a black experimenter. Across both experiments, concern with controlling bias and with being seen as biased was associated with the predicted distortion (slowing) in time perception.

Experiment one also explored if a temporary goal–to be egalitarian—might ameliorate the arousal people high in EMCP typically experience. There was no evidence to support this possibility. Regardless of their temporary goals, people high in EMCP showed the time slowing effect. Of course, it might be that the subtle manipulation of goals that we employed was not sufficient to overcome the more powerful arousal. Or it might simply be that providing an internally motivated goal does not remove arousal, but merely provides another goal that coincides with the external goal and its associated arousal. These issues should be explored in further research.

The basic phenomenon of interest–time slowing—was produced in two experiments. Those who were chronically concerned about appearing prejudiced as well as those who were supervised by a Black experimenter experienced time slowing in processing Black faces. Future research should also examine arousal as the mechanism of distortion in time perception during intergroup interactions more directly via physiological measures.

The impact of a time overestimation bias on human responding that requires timing as an essential element for action is another fruitful area for future research. These small scale perceptual differences could impact interaction, contributing to disparities that emerge in health care, criminal justice, and awkwardness demonstrated in everyday social interaction. Moskowitz, Olcaysoy Okten, Gooch, Moore-Berg, and Karpinski [50] have illustrated that how participants perform in a “shooter” task, where they must fire a simulated weapon at black versus white men, is impacted by their level of TPB as assessed by the discrimination task described above. Kenrick, Sinclair, Richeson, Verosky, and Lun [51] found that the speed with which a black man versus a white man appeared to be moving toward a white participant depended on arousal, speculating that time perception mediated the judgments of motion. It is already known that doctors terminate intake interviews with patients differentially as a function of the patient’s race, and there is widespread concern in the United States that police officers initiate force differentially as a function of race. Does bias in the perception of the duration of these interactions or the speed of movement contribute to these disparities? This is another fruitful topic of exploration.

Finally, TPB should not be limited to white participants. An interaction among a white person and a black person is known to be arousing for the black individuals in the encounter as well [32]. For many black Americans such interactions introduce concerns about being the target of bias, or being treated in a stereotypical way. It also introduces stereotype threat, a form of arousal that emerges from fearing one may confirm the group stereotype through one’s actions in the encounter [52]. Thus, black participants, indeed any individuals who may experience arousal in an interaction, should experience bias to time perception.
